# Health Promotion Programs to Reduce Noncommunicable Diseases: A Call for Action in Kuwait

**DOI:** 10.3390/healthcare8030251

**Published:** 2020-08-03

**Authors:** Ahmad Salman, Eleni Tolma, Sungsoo Chun, Kennedy O. Sigodo, Adel Al-Hunayan

**Affiliations:** 1 Ministry of Health, Safat 13001, Kuwait; 2 Faculty of Public Health, Kuwait University, Safat 13110, Kuwait; eleni.tolma@ku.edu.kw; 3 United Nations Development Programme in Kuwait, Safat 13030, Kuwait; sungsoo.chun@undp.org; 4 Department of Public Health, Glasgow Caledonian University London, London E1 6PX, UK; Kennedy.Sigodo@gcu.ac.uk; 5 Faculty of Medicine, Kuwait University, Safat 13030, Kuwait; adel.hunayan@ku.edu.kw

**Keywords:** health promotion, public health, Kuwait, socioecological model, physical inactivity, obesity, smoking

## Abstract

Most public health issues in Kuwait are related to unhealthy behaviours. Research shows that behaviours are the result not only exclusively of personal choices but also of myriads of other social and environmental factors. Kuwait is one of the leading countries in obesity and tobacco use in the world. Cardiovascular diseases stemming from complications related to these and other risk factors are important health issues based on their morbidity and mortality implications. These risks are spread across society and affect the old as well as young boys. The serious gaps between Kuwait’s health-related needs and the existing policies to reduce public health risks in Kuwait create a significant obstacle to healthy behaviour change. Kuwait requires adequate laws, policies, regulations, activities, and programs to promote people’s health. The Socio-Ecological Model (SEM) has been used successfully in health promotion in various behavioural settings, including obesity, physical inactivity, and smoking. We propose the use of SEM as a planning framework in building sustainable health promotion programs in Kuwait while paying attention to other concepts such as systems thinking, authentic community participation, community capacity, policy development, public health infrastructure enhancement, health coaching, and equity.

## 1. Introduction

The World Health Organization (WHO) defines health promotion to be a process that enables people to increase control over their own health [1]. It not only focuses on the treatment and cure of diseases but also encompasses a wide range of social and environmental interventions that are designed to address and prevent the root causes of ill health [1]. To improve people’s health, effective social and environmental interventions must be established. This is consistent with the global recognition of the role of wider social determinants of health and the shift in focus from medicine to public health and health promotion in improving population health.

According to a landmark report [2], there are five major domains of determinants of health and contributors to premature death. As the author states, even if the United States (US), where the study took place, had an excellent medical care, only a small fraction of premature deaths could be prevented (10%). In fact, the major contribution to premature death is behavioural patterns (40%), followed by the individual’s genetics (30%), the social circumstances we live in (15%), and environmental exposure (5%). Interestingly enough, the two top behavioural causes of premature death are smoking and obesity coupled with inactivity [2]. Obesity is a reliable indicator to predict cardiovascular diseases, hypertension, colon cancer, stroke, and other noncommunicable diseases [3,4].

The above report also brought attention to the role of socioeconomic status or social class (which is a composite construct of income, total wealth, education, employment, and residential neighbourhood) in health outcomes. People of low socioeconomic status are less healthy and have lower life expectancy. Environmental factors such as polluted air and water, lack of space for physical activity, or dangerous neighbourhoods also contribute to premature death since people from lower socioeconomic strata are more likely to be exposed to these health-compromising conditions [2]. Therefore, the interrelatedness of economic issues and health outcomes shows the imperativeness of considering both intended and unintended impacts of developmental policies and actions on public health.

Health promotion offers pathways that connect local programs’ implementation to national policies with links in global financing, trade, and investment policy under the framework of sustainable development [5]. Therefore, it is critical to integrate health promotion into national sustainable development goals (SDGs) strategies and implementation plans [5]. The interlinkages across the SDGs provide a framework for action across multiple sectors to promote good governance, to build healthy cities and communities, to enhance health literacy, and to support social mobilization for health [5].

The State of Kuwait is an emirate which has been governed by the Al-Sabah family since the 18th Century [6]. Kuwait is located within the Middle Eastern region of Asia. Given the country’s location, it is one of the driest and hottest in the world, with summer temperatures ranging between 42–48 °C [7]. Due to Kuwait’s size, 100% of the population resides in urban areas [8]. There are six governorates in Kuwait: Al-Asimah (capital), Hawalli, Al-Farwaniya, Mubarak Al-Kabeer, Al-Ahmadi, and Al-Jahra [7]. The Kuwait population, as of December 2019, totalled 4.8 million [6]; 3.3 million expatriates were living within Kuwait, thus comprising 70% of the total population, which is similar to that in other Gulf Cooperation Council (GCC) countries [6]. Sixty-nine percent of the expatriate population are men [6]. Kuwait has a young population with 44% of the Kuwaiti population being aged under 20 years. Those aged between 25 and 34 years represent the largest group in society, comprising 26% of the total population, indicating a large working-age population [6]. The official language in Kuwait is Arabic; however, English is widely spoken given the high proportion of migrant workers [7]. Regarding religion, approximately 75% of the population follow a Muslim faith, followed by Christianity (18%) [7]. The remaining 7% comprise a range of religions, either specified or not.

## 2. The Burden of Noncommunicable Diseases (NCDs)

Life expectancy at birth for the total population was 78.6 years in 2020 [7]. When broken down by gender, it is clear that life expectancy for women is higher than men in Kuwait (80.2 years vs. 77.2 years) [7]. The primary cause of death in developed countries, including Kuwait, are related to noncommunicable diseases (NCDs) [9]. In 2016, 72% of deaths in Kuwait were attributed to NCDs [9]. Specifically, in 2015, the mortality rate due to diseases of the circulatory system was 58.6/100,000 followed by 21.7/100,000 people for neoplasms [10]. Moreover, in Kuwait as well as in other GCC countries, the prevalence of diabetes in 2019 is high (12.2%) in contrast to other wealthy countries such as USA where the prevalence rate is 10.8% [11], and the corresponding mortality rate in Kuwait in 2015 was 3.2/100,000 people [10]. In addition, close to a quarter of the population has two or more chronic diseases [12]. Finally, the death rates of the three major causes of death (i.e., diseases of the circulatory system, neoplasms, and traffic-related injuries) have remained the same or showed little change from 2011–2015 [10]. In this commentary, we will focus only on the diseases of the circulatory system and neoplasms since they have similar pathways of causation and possible solutions.

## 3. Unhealthy Behaviours

In Kuwait, we observe a similar pattern of behavioural causes of premature deaths as the one in the USA. According to a recent WHO report [9], the physical inactivity rate in 2016 reached 60% for males and 73% for females in Kuwait (Table 1). The obesity rates were also high for both males and females, with 33% for males and 44% for females. Finally, the tobacco smoking rate among males aged 15 years and above is relatively high, at 40% of the population.

As a result, the burden of Noncommunicable diseases (NCDs) is rapidly increasing in Kuwait, estimating to account for 72% of all deaths in 2016 [9]. Between the age of 30 and 70 years, the probability of mortality from any of cardiovascular disease, cancer, diabetes, or chronic respiratory disease is 17.4 per 1000 persons, being ranked at 75th in the world [13]. Let us take a closer look at the three top behavioural causes of premature death in Kuwait.

### 3.1. Physical Inactivity

Kuwait is a country with the highest prevalence of insufficient physical activity around the world. Sixty-seven percent of adults do not have sufficient physical activity [14]. The following country to Kuwait is Saudi Arabia, which has almost 14% less than Kuwait, at 53.1% prevalence of insufficient physical activities.

Based on a recent report published in Kuwait [15], some of the reasons for the high inactivity include social stigma associated with walking outdoors, inadequately designed pedestrian walkways and bicycle routes, limited parks and facilities for sporting events, sedentary work environment, and sedentary school environment (physical education is often optional and not viewed as important as other classes) [16,17].

### 3.2. Obesity

There are many causes of obesity, including unbalanced nutrition intake, overwhelmed stress, and insufficient exercise. Kuwait is the 11th country with the highest prevalence of global obesity (37.9%) [18]. Pacific Small Island countries, which historically have a high prevalence for obesity and similar ethnic groups and lifestyle patterns, contribute to the majority of the top 10 countries for obesity.

As a result of the high obesity rate, Kuwait’s mean Body Mass Index (BMI) is also the highest among the GCC countries [19]. Kuwait’s BMI is the largest at 29.6 (29.0–30.1) kg/m^2^, followed by Qatar’s at 29.2 (28.4–29.9) kg/m^2^, Oman’s at 26.3 (25.4–27.2) kg/m^2^, and Bahrain’s at 24.8 (23.7–25.8) kg/m^2^.

**Figure 1:**
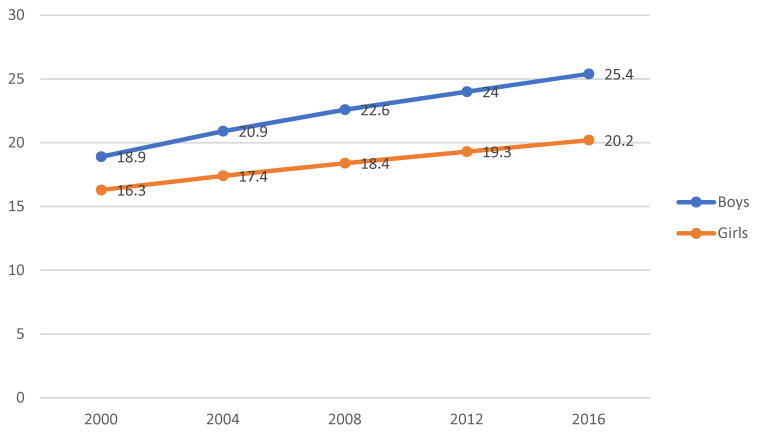
Trend of obesity of adolescents (aged 15–19). Source: Global Nutrition Report 2018 [20].

Obesity is an issue not only for adults in Kuwait but also children. Seven out of ten adolescents are obese or overweight [20]. The obesity and overweight rates among adolescents aged 15–19 years have dramatically increased in 2000–2016 [20]. A significant change concerns the prevalence of overweight and obese adolescents, which grew to 54.7%–70.1% of obese and overweight boys and 52.7%–60.1% of obese and overweight girls (2000–2016) [20]. Especially obesity rates among adolescents have been increasing from 18.9% in 2000 to 25.4% in 2016 among boys and 16.3%–20.2% among girls during the same period (Figure 1). Girls and women in Kuwait have higher rates of being overweight than their counterparts in the GCC countries.

Some of the reasons for the high obesity rate in Kuwait includes the lack of regulation on the amount of sugar and salt in foods and beverages as well as large gatherings among family members and their extended social networks, during which there is a lot of food consumption and little physical activity [16,17]. Moreover, only 18.6% of men and 14% of women consume a sufficient amount of vegetables and fruits per day while 76.8% of women and 70.3% of men consumed one cup of carbonated beverage daily [21].

### 3.3. Smoking

According to the WHO [22], smoking rates among Kuwait male adults have not changed and remained around 40% over the last two decades. The smoking prevalence rate among men aged 15 years in Kuwait (40%) is also the highest among the GCC countries, while the lowest prevalence is in Oman (16%). The highest reported smoking prevalence rate among women aged 15 years appears in Bahrain (5%) compared to (3%) in Kuwait, while the lowest rates are in Oman (0%).

The relatively high percentage of young smokers (13–15 years old) in Kuwait is alarming. A recent study conducted in 2016 shows that 24.2% of boys and 9.8% of girls in Kuwait are current tobacco users, whereas 19.4% of boys and 4.6% are current smokers [23].

Based on a cross-sectional survey conducted to study the epidemiology of smoking among Kuwaiti adults, the most common reasons for smoking among men and women were to relieve boredom and to feel relaxed [24]. When survey data were analysed separately for each gender, men gave considerably more weight to using smoking to help them concentrate at work (40%) and women using smoking to relieve anger and frustration (64%).

Despite efforts to ban smoking in public places and to impose tobacco taxes, there has been little change in smoking rates. The Kuwaiti government set a goal to reduce male adult smoking rates to 28% by 2025 after signing the WHO Framework Convention on Tobacco Control (FCTC) in June 2003 [22].

## 4. Current Efforts in Kuwait to Promote Health and to Address Noncommunicable Diseases (NCDs)

Kuwait is currently addressing the NCDs primarily via curative care or disease management and not through preventative measures [15]. There are many reasons for that including the lack of public health experts in the country, the narrow definition of public health in Kuwait, and the fact that the benefits of public health efforts may not be visible for decades compared to the benefits of curative measures. Therefore, the country does not see it necessary to invest in prevention [15].

However, Kuwait has taken some preventive measures toward reducing cardiovascular diseases including the restriction of salt used in all bread products by the Kuwait Flour Mills and Bakeries [25]. In addition, to improve knowledge on chronic diseases and prevention efforts, several educational training sessions have taken place among professionals, but there are very few related community outreach programs [15]. There are also various screening programs throughout the country such as Cancer Awareness Nation that promotes screening mammography and bowel screening; however, its uptake is very low [15].

In the fight against smoking, Kuwait has enacted smoke-free laws in certain facilities and places such as healthcare facilities, universities, and public transportation areas [23]. However, people can still smoke in governmental facilities, indoor offices and workplaces, restaurants, and cafes. There is also available treatment of tobacco dependence (nicotine replacement therapy) which is covered by the national health services program [23]. There are also several bans on tobacco advertising, promotion, and sponsorship via media. Finally, there are taxes imposed on most sold brands of cigarettes, with the current price of a pack of 20 cigarettes at 0.85 Kuwaiti dinars which is equivalent to 2.8 USD [23].

In curtailing diabetes, the Dasman Diabetes Institute was created, which is a not-for-profit organization. Its primary aim is to conduct research and, through the results, to inform policies related to the reduction of the prevalence of diabetes. Another positive recent development is the establishment of a Faculty of Public Health at the Kuwait University Health Sciences Center. The Faculty offers a four-year undergraduate program in four tracks (public health practice, health care management, health research, and community health development) as well as professional Master of Public Health degrees offering specialized training in community health, policy development, epidemiology, and public health practice. It is expected that graduates of the program will be able to work in areas in public health or related professional areas and be able to apply the skills and knowledge they have acquired through the courses in the promotion of health in Kuwait.

In terms of Kuwait’s healthcare infrastructure, one of its prominent features is the establishment of 94 governmental primary health care clinics, besides the 14 governmental hospitals, and 81 diabetic clinics [10]. The primary health care clinics provide a variety of health care services including preventative care, general health, and childcare. For all residents in Kuwait (nationals and expatriates), these clinics’ services are provided at a low cost [15].

The ongoing collaborations with the United Nations to contextualise global interventions to suit Kuwait are essential for the national health efforts. The UN agencies frequently make recommendations to assist the government of Kuwait in developing a more rigorous strategy to reduce the prevalence of NCDs and to improve health promotion in Kuwait [26]. The WHO recommends that Kuwait consider adopting a more systematic cardiovascular risk stratification for early detection of cardiovascular diseases using WHO Global HEARTS initiative tools [26]. It has also suggested that Kuwait align national efforts to prevent and treat cancer with new emerging global and regional WHO guidance on cancer prevention and control [27]. The Joint Mission of the United Nations Interagency Task Force on the Prevention and Control of NCDs (UNIATF) recommends that Kuwait adopt the Healthy City Initiatives that address health issues associated with pollution by planning new cities in ways that focus on minimizing environmental hazards as part of providing a healthy environment to live, work, and play [26].

The 9th global conference in Shanghai in 2016, titled “Promoting health in the Sustainable Development Goals: Health for all and all for health”, highlighted the critical links between promoting health and the 2030 Agenda for Sustainable Development [5]. The Shanghai Declaration provides a framework through which governments can utilize the transformational potential of health promotion [28]. It is critical to integrate health promotion into national sustainable development goals (SDGs) strategies and implementation plans [5]. The interlinkages across the SDGs provide a framework for action across multiple sectors to promote good governance, to build healthy cities and communities, to enhance health literacy, and to support social mobilization for health [5].

WHO recommends focusing on the five areas of work for health promotion: good governance, health literacy, healthy cities, health-promoting schools, and social mobilization [28]. Evidence-based health promotion uses information derived from formal research and systematic investigation to identify causes and continuing factors to health needs and the most effective health promotion actions to address these in given contexts and populations [29].

## 5. Moving Forward—Recommendations for Addressing NCDs in Kuwait through the Lens of Health Promotion and Socioecological Model (SEM)

Health promotion, as said at the beginning, is the process of enabling individuals and communities to gain control over their determinants of health and to improve their quality of lives [1]. Health promotion is an empowering process that helps individuals make healthy lifestyle choices once they are given the necessary information and skills within a supportive social, economic, and political environment. In other words, individuals can modify their existing unhealthy behaviours through one-on-one counselling; however, they may not be able to sustain the new behaviour over a longer period of time unless there are supportive policies/programs or environmental changes [30]. Another reason for using an ecological framework in health promotion is that, by using population-based interventions through new technological advances, communication strategies, or social media (provided that there is no digital divide), we might be able to reach large segments of the population who are at greater risk of developing NCDs and yet not having enough social or economic resources. Working with underserved populations might require more face-to-face, interpersonal, or local community-based interventions [30].

Health promotion is also about empowering communities and transforming them into healthy communities by establishing new social or cultural norms and by enacting supportive policies and environmental changes. Empowering communities is achieved by building community capacity [31]. Community capacity is the community’s potential or ability to address health or social issues. It is synonymous to community readiness [31]. There are many dimensions in building community capacity including leadership, skills, community’s history, resources including social capital, social networks, sense of community and pride, community’s values, and true participation of the individuals comprising the community [31]. Community or public participation is a key factor of building community capacity.

There are many forms of participation, starting from the nonparticipation level, where the local health department initiates and directs the community to act (top-down approach); to the middle approach, where the health department asks community members their input via advisory boards or focus group research; and the full participation or citizen power approach, where the community members initiate and direct the public health effort (bottom-up approach) [32]. True participation refers to the latter approach where community members work with the health department to identify issues that are salient to them and to identify the solutions to these issues. The role of the health department is to support the process and to provide the resources needed to the community members. A very good example of true community participation is the Turning Point Initiative, where governmental and private entities through partnerships develop system changes (i.e., a modified program or policies) that enable the people to live in healthy conditions [33]. We understand that promoting true community participation in Kuwait via the Ministry of Health might not be realistic because of existing cultural norms; however, we recommend that the middle form of community participation should be encouraged in the development of health promotion programs.

Participation can be achieved via community outreach, by working closely with different civil society organizations, and by mediating structures (e.g., professional organizations). In Kuwait, there are close to 100 civil society organizations and professional associations representing many sectors of society outside health, including the arts, sports, or business sectors. For example, the arts can help in promoting health by increasing awareness and relaying health education messages, by getting people involved in a health-related event while at the same time having fun, by learning more about the community such as conducting needs assessments, by attracting attention to an issue, and by promoting community building [34]. Another example stemming from the business world is the promotion of worksite health and wellness. Studies have shown that worksite health promotion programs can reduce sick leave absenteeism by 27% and can reduce health care costs by 26% [35]. One example of a program promoting wellness in communities including the business sector is the “Certified Healthy Business Program” [36], during which businesses are committed to supporting healthy choices through environmental and policy changes. There are several aspects that need to be addressed in order to receive certification including a strategic plan for a health promotion program, conducting an employee health risk appraisals/assessment, and having an active diverse health promotion committee.

Another way to promote community participation is in the form of coalitions, where different groups of people and civil society organizations collectively advocate for a common public health issue [37]. There are mixed reviews about the effectiveness of coalitions in promoting health [38]; however, they can definitely be another tool in promoting health in conjunction with other strategies.

A form of community participation was used in 2017 when the Faculty of Public Health led a workshop on identifying the priorities for intervention addressing the NCDs in Kuwait, the major barriers that challenge public health efforts to prevention and control of NCDs, and possible solutions [39]. The workshop was attended by 66 stakeholders in Kuwait, representing governmental as well as private entities. The researchers used a modified Delphi technique. The stakeholders ranked obesity as the main public health priority, lack of national vision of the development of a public health system as the most important barrier, and investment in health care prevention as the top solution.

Evidence shows that the use of theories enhances the designing, implementation, and effectiveness of health promotion interventions [40]. This study posits the Social Ecological Model (SEM) as a helpful planning framework [40,41]. The SEM is a theory-based framework for understanding, exploring, and addressing the social determinants of health at many levels [42]. It provides knowledge of how the social determinants of health influence and maintain health and health-related issues. Also, it helps identify promising points of intervention and provides a better understanding of how social problems are produced and sustained within and across the various subsystems [42]. The model demonstrates that behaviour is the result of knowledge, values, and attitudes of individuals as well as social influences, including the people with whom they associate, the organizations to which they belong, and the communities in which they live [42]. The health promotion programs based on the SEM are the strategic alignment of policy and services across the continuum of population health needs, including the design of effective health promotion and disease prevention and control strategies.

The SEM has been used in multilevel approaches to areas such as in public health promotion. Multilevel interventions are strategies that aim to change behaviours and to address health outcomes by affecting more than on the level of influence within the SEM [43]. It is also important to note that each level of analysis is embedded in a system characterized by reciprocal causality. For example, individuals (intrapersonal level) are affected by the social networks they live in (interpersonal level), while at the same time, the individual’s personal characteristics affect the function of the network. This suggests that intervention at one level can produce an effect at another level [44]. Golden and Earp identified the ecological levels that health promotion programs target from 157 intervention articles published in the scientific journals from 1989 to 2008 using the Medline database [45]. Their review found that most interventions focused on the individual or interpersonal levels and that most successful applications were done to promote physical activity and healthy nutrition in school settings. Very few interventions focused on the policy or community levels and the authors explained that addressing all levels of the ecological model might be difficult to implement and evaluate.

SEM framework can be applied in reducing obesity. Obesity is the product of a complicated system of networks, relevant factors, and their interdependencies that determine the condition of obesity for an individual or a group of people. This system includes causal links and feedback loops. Viewing obesity from a system thinking perspective—an expanded model of SEM—can help practitioners and policymakers decide where to most effectively intervene in the system by taking in consideration leverage points, feedback loops, and causal cascades.

Based on a report developed in the United Kingdom (UK) [46], the obesity system map includes many interconnected factors that fall into the following categories: (a) media (e.g., media consumption); (b) economics (e.g., cost of ingredients); (c) psychological status (e.g., stress); (d) social status (e.g., parental control); (e) food (e.g., force of dietary habits); (f) activity (e.g., level of domestic activity); (g) infrastructure (e.g., walkability of living environment); (h) developmental factors (e.g., quality and quantity of breastfeeding); (i) biological factors (e.g., genetic or epigenetic predisposition to obesity); and (j) medical factors (e.g., reliance on medical interventions). The map also includes leverage points for possible interventions. For example, in relation to educational intervention, health educators or health promoters can focus on promoting food literacy, on educating the public about portion sizes, or on addressing social acceptability of fatness in the society.

Another group of leverage points concerns the purchasing power of the consumers. Some possible points for intervention include efforts to increase efficiency of production, to enhance the nutritional quality of food and drinks, or to decrease the cost of physical activity. Therefore, one of the first recommendations for Kuwait’s policy makers is to view obesity as a system of interconnected factors and not merely the individual’s responsibility for becoming obese as referred to as “victim blaming”, a negative consequence of traditional health promotion [47]. The policymakers can in fact adopt the existing map and can adapt it to Kuwaiti society or can develop their own obesity map with the use of qualitative and quantitative research.

Once policymakers agree that obesity is a multicausal problem, then they can start planning health promotion interventions. A popular venue of intervention in regard to obesity is schools. School-based interventions targeting children, youths, and their parents have been used extensively and successfully in obesity prevention [48,49]. Schools are a preferred setting for interventions because children represent an easily accessible captive audience that is still young enough to form new behaviours. We recommend that the Ministry of Health in collaboration with the Ministries of Education and Youth take a leading role and develop a plan of action based on the SEM and on evidence-based health promotion interventions to promote healthy nutrition practices as well as physical activity at schools. One good starting point is the Whole School, Whole Community, Whole School Model (WSCC). The WSCC model includes 10 components that collectively aim to ensure that each student is safe and healthy and learns about and practices a healthy lifestyle [50].

Focusing on the SEM’s level of policy development in the area of obesity, it was interesting to find out that Kuwait’s neighbour country, Saudi Arabia, has recently enacted a Healthy Food Strategy, which includes limiting trans fatty acids to 2% for fats and oils and to 5% in other foods; reducing sugar, salt, and fat in food products; requiring calorie labelling in restaurants and cafes; and improving nutrition surveillance [51]. The Saudi government used a multisectorial approach to pass these policies including partnerships with the industry, academia, and other civil society organizations. Kuwait can learn from Saudi Arabia’s experience in passing similar bans besides the reduction of salt in baked foods.

Another global initiative or movement in line with the socioecological approach to health as well as the principle of community participation is the concept of the “Healthy City” or “Healthy Community” movement. The respective movement started in Europe in 1986, and since then, it had spread throughout the world [52]. The underlying premise of this initiative is that living conditions including physical, social, and economic conditions influence the citizen’s health outcomes and quality of life. The “Healthy City” initiative is a citizen-led program, during which citizens identify health problems; analyse the causes and factors; and, in collaboration with local authorities, develop a city plan for action. In Kuwait, there is already one accredited “Healthy City” and 8 more that have been registered and are waiting evaluation [53].

Kuwait has an overall good-quality primary health care system with many clinics throughout the country, where most residents have free or low-cost access to it. Unfortunately, most emphasis is put on disease management and not on prevention. On the other hand, the existence of this primary health care system can be seen as an asset in the community which can be strengthened. One way to strengthen this asset is by changing the emphasis from tertiary prevention to primary and secondary prevention. This can be done by encouraging physicians and the patients to conduct annual health check-up examinations which will include a physical examination, counselling, clinical tests, and screening tests (annual blood pressure tests and cancer screening tests) while at the same time promoting health coaching or a patient-centred approach to health decision-making.

Conducting annual health check-up examinations can be implemented through policy development, coupled with social marketing campaigns aiming to change the current public’s perception that a person needs to visit the doctor’s office only when he/she is sick rather than to find out if there are any underlying diseases even when she/he is not experiencing any symptoms.

Health coaching can also be a valuable tool to health care personnel in promoting preventative care. Health coaching is defined as “helping patients gain the knowledge, skills, tools, and confidence to become active participants in their care so that they can reach their self-identified health goals” [54]. Physicians themselves can do health coaching; however, it is also done by health educators, nurses, or social workers who serve as the “bridge” between the patient and the physician. It requires extensive training in communication skills. Evaluation of the health coaching practice in the UK found higher patient compliance, reductions in episodes of care, improved care quality, potential reduction of waiting times, and less waste of unnecessary medication [55].

Before we move to some overall recommendations, we cannot overlook the importance of prevention on tobacco use in Kuwait. We acknowledge that Kuwait has taken some preventative measures. However, those measures have not proven sufficient to stop smoking [23]. We recommend an expansion of smoke-free laws in other places such as governmental facilities, indoor offices and workplaces, and restaurants. We also recommend an anti-tobacco mass media campaign to support such policies and public opinion surveys on tobacco use. Finally, we also need to remember that the “war” against tobacco use is a long one. It took the US more than 50 years to change the public’s perceptions toward smoking so that smokers are seen as weak willed and addicted. This change in cultural norms occurred through the lens of the SEM (i.e., educating on a personal basis the harmful effects of smoking while working with families and social networks) and by utilizing multiple other systemic interventions (e.g., bans on smoking in public spaces, rises in the cigarette taxes, anti-tobacco advertising, and health communication campaigns). All these interventions synergistically contributed to the decrease in tobacco use in the US [44].

## 6. Call for Actions: National-Level Actions for Health Promotion

The commentary makes a call for action to quickly transform increasingly unhealthy behaviour and unhealthy status into healthy behaviour and good health status. The following are some specific recommendations.

First, invest in the training of public health professionals specialized in health promotion. Health promotion is considered an “art and a science” [47]. The “art” part comes from the fact that health promoters need to appropriately apply theoretical frameworks of both levels: the individual/behavioural and community settings across many behaviours, populations, and contexts. It also refers to the ability to design health promotion programs, to implement them, and to evaluate them. These skills are the product of both acquired knowledge and experience. There is a public misconception that health promotion and education are mostly about “raising awareness”, whereas health promotion is clearly more than that. Raising awareness is important to changing behaviour but is not sufficient [56]. Second, build surveillance systems that monitor the prevalence of healthy or unhealthy behaviours. The implementation of the WHO STEP-wise approach to surveillance (STEPS) studies is a step toward that direction [21]; however, more current data are needed on a continuous basis so that behavioural gaps are identified within different segments of the population. Third, strengthen existing community-based programs like Cancer Awareness Nation which is focusing on screening. Through proper evaluation, program implementers can find out why the uptake is so low and can identify ways to promote participation. Finally and most importantly, promote equity. Equity is closely related to the concept of social determinants of health. In other words, the social environment affects people’s health and welfare [57]. There are 12 dimensions of the social environment: economy, employment, education, politics, environment, housing, medical, governmental, public health, psychosocial, behavioural and transport [58]. To achieve equity among all its residents, Kuwait has to address each dimension, which requires a multidisciplinary approach. Health promotion can assist in this effort by addressing various aspects including the behavioural one through the development of surveillance behavioural systems for all residents in Kuwait.

## 7. Conclusions

Health promotion is the only pathway toward building a healthy Kuwait, by enabling and empowering its people and communities to control their health. However, there is no single policy or program toward promoting health. In line with the socioecological approach to health, health promotion requires a package of programs and policies, considering key contents of the contributing factors, programs and activities, targets, communication methods, and participant groups at each level of the socioecology model.

## Figures and Tables

**Table 1:** Risk factors of noncommunicable diseases (adults aged 18+, 2016, %).

Risk Factors	Male	Female	Total
Physical inactivity	60.0	73.0	65.0
Current tobacco smoking (aged 15+)	40.0	3.0	24.0
Obesity	33.0	44.0	37.0

Source: WHO Kuwait noncommunicable diseases profile [9].
